# Determinants of Treatment Outcome, Follow-Up, and Abstinence Rates Among Patients With Alcohol Use Disorder: A Prospective Study

**DOI:** 10.7759/cureus.31356

**Published:** 2022-11-11

**Authors:** Madhura GC, Avudaiappan Sankaran, Karthick Subramanian, Eswaran Subramanian

**Affiliations:** 1 Psychiatry, Mahatma Gandhi Medical College and Research Institute, Pondicherry, IND

**Keywords:** impulsivity, follow up, abstinence, relapse, alcohol use disorder

## Abstract

Background

The treatment of alcohol use problems needs to have a multidimensional approach for early recovery and better outcome.

Objective

The study aimed to study the socio-demographic, clinical, and psychological factors associated with the three-month clinical outcomes among patients with alcohol use disorder treated in a tertiary care general hospital psychiatric unit in southern India.

Methods

This is a prospective three-month follow-up study. Patients fulfilling the Diagnostic and Statistical Manual of Mental Disorders (DSM)-5 criteria for alcohol use disorder were selected for the study. The baseline severity of alcohol use was assessed by the Severity of Alcohol Dependence Questionnaire, alcohol craving by Alcohol Craving Questionnaire (ACQ), impulsivity by Barratt Impulsiveness Scale, anxiety and depression by Hospital Anxiety and Depression Scale and personality traits by Personality inventory for DSM-5. Craving was assessed at 2, 4, 8 and 12 weeks post-discharge.

Results

A total of 110 patients participated in the study. After 3 months of follow-up, 75.5% of the patients remained abstinent throughout the follow-up period. The sample was divided into two groups based on the abstinent status at 12 weeks. Both the abstinent and relapsed users were compared based on socio-demographic, marital, family, illness-related, alcohol-usage, and psychological characteristics, the baseline and follow-up craving indices. There were no statistically significant differences between the two groups based on socio-demographic characteristics. Compared to the relapsed group, the abstinent individuals displayed increased adherence to follow-up visits and attended more frequent individual therapy sessions, however, there were no significant differences between the two groups in the number of group therapy sessions attended. Also, the abstinent group demonstrated lower subjective craving at both the baseline and during follow-up. The relapsed users had reported a significantly greater proportion of history of suicide in the family. Further correlational analyses were done to find the direction of associations between psychological characteristics and alcohol-usage characteristics. High attentional impulsivity and motor impulsivity were associated with early age of first use. High attentional impulsivity, motor impulsivity and impulsive planning were associated with early age of dependence. Early age of dependence was associated with increased levels of antagonism and psychoticism. Younger onset of alcohol use was associated with increased levels of craving at baseline. The baseline alcohol use severity and the baseline craving towards alcohol were positively correlated with follow-up craving indices as per ACQ and craving index (CI) scales.

Conclusion

The patients who were abstinent displayed reduced subjective craving towards alcohol at baseline and were more adherent in attending individual and group therapy sessions. Among the relapsed users, most relapsed before four weeks and had a family history of alcohol dependence, family history of psychosis, and suicide, which is indicative of high genetic loading of substance abuse leading to early initiation and early onset of alcohol dependence. The effects of genetic loading could be possibly mediated by traits of high impulsivity, psychoticism, and antagonism.

## Introduction

Alcohol use disorders have long been recognized to have remitting and relapsing courses. According to Durazzo and Meyerhof, irrespective of the type of intervention employed, at least 60% of treated individuals for an alcohol use disorder relapse to a period of hazardous alcohol consumption, typically within six months of treatment [1]. Relapse is a multifaceted problem with reasons being found in biological (craving, depression), sociocultural (family support), and psychological (personality) domains. Identifying patient characteristics that have putative associations with relapse risk after de-addiction treatment is important for developing specialized or customized treatment programs for those at risk [2-4].

Previously Kar et al. addressed the question of the factors influencing the short-term outcome of alcohol dependence among 60 patients in the Indian setting. They found that a positive outcome was seen in 55% of patients. However, they could find no association between any of the sociodemographic or clinical variables they studied [5]. A similar cross-sectional study done by Sureshkumar et al., however, found that a family history of substance dependence and past history of ≥2 relapses, younger age of onset of dependence, and a shorter time to develop dependence were significantly associated with relapse [2].

However, prospective naturalistic follow-up studies incorporating data about illness characteristics, adherence to pharmacotherapy, and psychotherapy are very few in Indian settings. Hence, we intended to prospectively study the socio-demographic, clinical, and psychological factors associated with the three-month clinical outcomes among patients with alcohol use disorder treated in a tertiary care general hospital psychiatric unit in southern India.

## Materials and methods

Study setting, design, and participants

This is a prospective three-month follow-up study. After obtaining approval from the institutional ethical committee (#MGMCRI/Res/01/2019/106/IHEC/039), patients fulfilling the Diagnostic and Statistical Manual of Mental Disorders-5 (DSM-5) criteria for alcohol use disorder and have been admitted to the department of psychiatry were selected for the study after obtaining informed consent. Patients with substance dependence other than alcohol or nicotine, diagnosed with a primary psychiatric illness other than Substance Use Disorder (SUD), and those with acute psychiatric disturbance or cognitive impairment were excluded from the study. The sample size calculated was 95. Out of that, 10% dropouts were predicted, so the sample size was calculated as 106, and we had taken a sample size of 110.

Study instruments and assessment

Socio-demographic data and other relevant clinical data, including past and family history of psychiatric illnesses and substance abuse, were explored using a semi-structured proforma. Standard weighted questionnaires were used to assess outcomes of interest as follows: the Severity of Alcohol Dependence Questionnaire (SADQ) was used to assess the baseline severity of alcohol use; alcohol craving was assessed using Alcohol Craving Questionnaire - Short Form (ACQ); impulsivity was assessed using Barratt Impulsiveness Scale (BIS); and anxiety and depression by Hospital Anxiety and Depression Scale (HADS). Personality traits were assessed by the Personality Inventory for DSM-5 (PID-5).

Barratt Impulsiveness Scale (BIS)

BIS-11, a 30-item questionnaire, is used to assess three facets of impulsivity: (1) attentional impulsivity, (the lack of ability to concentrate or focus attention), (2) motor impulsivity (acting without thinking), and (3) non-planning impulsivity (the absence of future planning and forethought). BIS is a self-report questionnaire with internal consistency coefficients that range from 0.79 to 0.83 for various populations [6].

Severity of Alcohol Dependence Questionnaire (SADQ)

The SADQ is a short, easy-to-complete, self-administered, 20-item questionnaire designed to measure the severity of dependence on alcohol as formulated by Edwards & Gross (1976) and Edwards (1978) [7]. There are five subscales with four items in each: Physical Withdrawal, Affective Withdrawal, Withdrawal Relief Drinking, Alcohol Consumption, and Rapidity of Reinstatement. Each item is scored on a 4-point scale, ranging from “Almost Never” to “Nearly Always,” resulting in a corresponding score of 0 to 3. Thus, the total score ranges between 0 to 60. Cronbach's alpha of 0.98 indicated a high internal reliability. It has good test-retest reliability and content and criterion validity.

Hospital Anxiety and Depression Scale (HADS)

The Hospital Anxiety and Depression Scale (HADS) was originally developed by Zigmond and Snaith to assess the amount of anxiety and depression that a person experiences. HADS is a 14-item scale. Seven of the items were related to anxiety, and seven were related to depression [8]. Higher scores indicate greater distress.

Personality Inventory for DSM-5- Brief Form (PID-5)

The adult scale of PID-5 has a 25-item self-assessed personality trait assessment scale for adults aged 18 and above. It assesses five personality trait domains, including negative affect, detachment, antagonism, disinhibition, and psychoticism. Each item is rated on a four-point scale (that is, 0=very false or often false, 1=sometimes or somewhat false, 2=sometimes or somewhat true, 3=very true or often true). The mean value of Cronbach's alpha coefficients reached 0.73 on the facets and 0.84 for domains. The overall scores range from 0 to 75, with higher scores indicating greater overall personality dysfunction. [9].

Alcohol Craving Questionnaire - Short Form - Revised (ACQ-SF)

The ACQ-SF contains 12 items from the 47-item Alcohol Craving Questionnaire (ACQ-NOW) developed to measure craving for alcohol among alcohol users in the current context. The ACQ-SF contains 12 items strongly correlated with the four subscales and total ACQ [10]. The Cronbach’s alpha for this scale ranged between 0.77 and 0.86. Higher scores indicate greater severity of craving [11]. 

Study procedure

The patients then received combined pharmacological and psychological interventions, which included treatment for alcohol detoxification and withdrawal symptoms followed by intensive group and/or individual therapy.

On average, a patient would spend a minimum duration of three weeks in the hospital for assessment, evaluation, detoxification, and inducting into group therapy sessions. The group therapy consisted of a group of 12-15 patients, moderated by a psychologist and a psychiatric social worker. The group therapy followed the psycho-educational design addressing the general impact of substance use, interaction with mental health, relapse prevention techniques, and lifestyle skills. A total of six therapy sessions were carried out in the study at the rate of one session every two weeks covering hospital stay and the follow-up period. Since the study was conducted during the COVID-19-related lockdown period with limited logistic support and social distancing norms, a considerable proportion of patients were assessed individually and provided follow-up individual therapy. Both individual and group therapy sessions were conducted using principles of motivational enhancement therapy (MET). Whenever physical visits were disrupted during follow-up, telephonic counseling services were provided from the study center.

The patient, after discharge, was advised to attend the Substance Use Disorder (SUD) clinic held every fortnight at the treatment center. The following primary outcomes were assessed at two weeks, four weeks, eight weeks, and 12 weeks post-discharge:

Lapse - Occasional (once for a period of one day) alcohol intake but not reaching the level of a regular drinking pattern.

Relapse - Resumption of previous drinking pattern that requires re-admission.

Abstinence (assessed only at the end of the study) - The patient had not consumed any alcohol for a duration of three months.

During follow-up, along with the primary outcome, subjective craving towards alcohol was also assessed. If the patient skipped physical visits to the SUD clinic due to lockdown-related logistic difficulties, such patients were approached through telephonic helpline services at their convenience, and the outcome measures were assessed over the phone along with corroboration from the primary caregiver living with the patient. In case the patient has relapsed prior to the 12 weeks period, it was taken as the study endpoint for that patient, and further assessments of outcome measures were deferred for such patients. However, efforts were made to make the patient come for further treatment. Patients with lapse continued to be in the study and underwent treatment as usual.

Statistical analysis

Data analysis was performed using Microsoft Excel software (Microsoft Corporation, Redmond, USA) and Statistical Package for Social Sciences (SPSS for Windows, Version 17.0., SPSS Inc., Chicago, USA). For descriptive analyses, mean and standard deviation were used to depict the distribution of continuous variables, and frequency and percentages were used to depict the distribution of the data pertaining to the categorical variables. For inferential analysis, based on the study objectives, comparison tests were adopted among various groups and sub-groups in the sample. A normality check of the data was performed using the Kolmogorov-Smirnov test. Comparison analyses for continuous variables were done in the following manner: for continuous variables following normal distribution between two groups, an independent sample t-test was used; for continuous variables following normal distribution between three or more groups, one-way ANOVA was used. A repeated measures ANOVA was used for the comparison of follow-up ACQ and CI scores between abstinent and relapsed users. Bonferroni correction was used for multiple pair-wise comparisons in ANOVA and RM-ANOVA. Pearson correlation test was used for correlational analyses between two continuous variables following a normal distribution. For finding the association between two or more categorical variables, the Chi-Square test was used. Wherever the cell values were less than five, Fisher Exact test was used. Effect sizes for significant differences between groups were computed using Cohen’s d (independent t-test), phi (Chi-square), and partial eta squared (ANOVA). For all inferential analyses, the statistical significance was set at p-value < 0.05.

## Results

Baseline socio-demographic, personal, and family characteristics

The study group consisted of 109 male patients (99.1%) and one female patient. Most of the patients were in their forties, educated up to 5th to 10th standard, and hailed from an urban background. The majority of the subjects (37.3%) belonged to upper-lower socioeconomic status. Most of the patients were married (74.5%), and a majority of the married patients (43.6%) did not experience marital conflicts. A majority (61.8%) lived in nuclear families, and most patients (63.6%) had a positive family history of substance use. Around half of the sample (54.5%) were manual laborers. The results are shown in Table 1.

**Table 1:** Sociodemographic variables *p<0.05 statistically significant

Variable		Abstinent (n=85), Frequency (%) or Mean (SD) or Mean Rank (Sum)	Relapsed (n=25), Frequency (%) or Mean (SD) or Mean Rank (Sum)	Test statistic (p-value)	Effect size
Age (in years)		40.92 (±11.37)	40.48 (±8.55)	t= 0.178 (0.859)	d=0.0437
Age group	18-30 years	18 (21.2%)	3 (12.0%)	c^2 ^= 5.526 (0.237)	NA
	31-40 years	22 (25.9%)	8 (32.0%)		
	41-50 years	29 (34.1%)	13 (52.0%)		
	51-64 years	14 (16.5%)	1 (4%)		
	65 & above	2 (2.4%)	0		
Education level	Illiterate	6 (7.1%)	4 (16%)	c^2 ^= 5.687 (0.224)	
	5th to 10th	52 (61.2%)	16 (64.0%)		
	12th	12 (14.1%)	0		
	Graduate	14 (16.5%)	5 (20%)		
	Postgraduate	1 (1.2%)	0		
Domicile	Rural	20 (23.5%)	9 (36%)	c^2 ^= 2.569 (0.277)	
	Urban	44 (51.8%)	13 (52%)		
	Semi-urban	21 (24.7%)	3 (12.0%)		
Socio economic status	Upper	3 (3.5%)	0	c^2 ^= 2.367 (0.669)	
	Upper-middle	10 (11.8%)	5 (20%)		
	Lower-middle	30 (35.3%)	8 (32%)		
	Upper-lower	31 (36.5%)	10 (40%)		
	Lower	11 (12.9%)	2 (8.0%)		
Marital status	Unmarried	23 (27.1%)	5 (20%)	c^2 ^= 0.507 (0.476)	
	Married	62 (72.9%)	20 (80%)		
Duration of marriage (in years)		13.43 (±12.59)	13.64 (±11.28)	t= -0.075 (0.940)	
Marital conflict	Yes	31 (36.5%)	3 (12%)	c^2 ^= 8.522 (0.014) *	φ = 0.278 Medium effect
	No	31 (36.5%)	17 (68%)		
	Not applicable	23 (27.1%)	5 (20%)		
Family history of substance use	Yes	53 (62.4%)	17 (68%)	c^2 ^= 0.266 (0.606)	NA
	No	32 (37.6%)	8 (32%)		
Family history of psychosis	Yes	1 (1.2%)	4 (16%)	c^2 ^= 9.784 (0.002) *	φ = 0.298 Medium effect
	No	84 (98.8%)	21 (84%)		
Family history of suicide	Yes	4 (4.7%)	4 (16%)	c^2 ^= 3.654 (0.05) *	φ = 0.182 Mild effect
	No	81 (95.3%)	21 (84%)		
Age of first use (in years)		20.78 (±6.98)	20.24 (±5.39)	t= 0.354 (0.724)	NA
Age of dependence (in years)		27.06 (±7.93)	24.84 (±6.05)	t= 1.291 (0.199)	
Time to dependence (in years)		6.28 (±6.09)	4.60 (±2.48)	t= 1.344 (0.182)	
Previous abstinent period (in months)		58.29 (±4954.50)	46.02 (±1150.50)	U= 825.5 (0.084)	
Lifetime abstinence	Partial abstinence	47 (55.3%)	10 (40%)	c^2 ^= 1.814 (0.404)	NA
	Complete abstinence	25 (29.4%)	10 (40%)		
	Continuous use	13 (15.3%)	5 (20%)		
IU per day		16.32 (±9.82)	17.00 (±7.92)	t= -0.318 (0.751)	NA
SADQ total		22.19 (±14.89)	28.32 (±14.88)	t= -1.810 (0.073)	
Comorbid tobacco use	Yes	28 (32.9%)	4 (16%)	c^2 ^= 2.688 (0.101)	NA
	No	57 (67.1%)	21 (84%)		

Alcohol usage characteristics

In our sample, most patients had their first use of alcohol around 20.66 (± 6.63) years and achieved dependence by 26.56 (±7.57) years. The average time taken to become dependent on alcohol by the patients was 5.90 (± 5.52) years. A majority of the sample (61.8%) remained untreated for their alcohol use disorder before enrolment into the study.

Comparison between abstinent and relapsed users

Among patients who were hospitalized, the mean duration of hospital stay is 22.60 (±8.49) days. During hospitalization and during subsequent discharge, a patient attended the SUD clinic for an average of 2.15 (± 2.39) visits, involving 3.42 (±2.24) group therapy sessions and 2.68 (±1.54) individual therapy sessions. During follow-up, 33 (30%) patients received anti-craving drugs.

During follow-up at the end of 12 weeks, 85 (77.27%) remained abstinent and 25 (22.72%) relapsed. The sample was divided into two groups, namely abstinent and relapsed groups. Baseline socio-demographic, clinical, alcohol-usage, and psychological characteristics were compared between the two groups.

Socio-Demographic and Clinical Differences

Both abstinent and relapsed users were similar across age groups, education levels, domicile, socioeconomic status, and marital status. Family history of psychosis (p=0.002) and suicide (p=0.05) were more common in the relapsed group at the end of three months. The proportion of marital conflict was greater among abstinent users than the relapsed users (p=0.014). The family history of suicide (φ = 0.182) had a mild effect, whereas marital conflict (φ = 0.278) and family history of psychosis (φ = 0.298) had a moderate effect in causing the between-group differences among abstinent and relapsed users.

There were no statistical differences between the two groups based on past medical and psychiatric characteristics such as a past history of seizures, delirium, induced psychosis, presence of other psychiatric or physical illnesses, past treatment characteristics, and duration of previous hospitalizations.

Alcohol Usage Characteristics

The age of initiation of alcohol (p=0.724), the age of developing dependence towards alcohol (p=0.199), the time required to become dependent on alcohol (p=0.182), and the period spent in abstinence (p=0.084) were similar across the two groups. The severity of alcohol use was similar across the two groups (p=0.073).

Psychological Attributes

There were no statistically significant differences between the two groups based on psychological characteristics such as impulsivity measured by BIS, depression and anxiety measured by HADS, and personality traits (negativity, detachment, antagonism, disinhibition, and psychoticism) as measured by PID.

Treatment Compliance, Abstinence, and Relapse

During the follow-up period, compared to the relapsed users, the abstinent group had a greater frequency of SUDC visits (p<0.001) and had more frequent individual therapy sessions (p=0.001). There were no significant differences between the two groups in the number of group therapy sessions attended (Table 2). Both at baseline and during the second week of follow-up, the patients in the abstinent group had lesser cravings, as shown by ACQ scores and CI scores than the relapsed group. However, the effects due to the time interval were mild to explain the within-group differences (partial eta, η² p = 0.117) (Table 3).

**Table 2:** Comparison of treatment characteristics between abstinent and relapsed users *p<0.05 statistically significant; SUDC: substance use disorder clinic

Parameter	Abstinent (85), Frequency (%) or Mean (SD)	Relapsed (25), Frequency (%) or Mean (SD)	Test statistic (p-value)	Effect size (Cohen’s d)
Anti-craving drug administration				
Yes	26 (30.6%)	7 (28%)	c^2 ^= 0.062 (0.804)	NA
No	59 (69.4%)	18 (72%)		
Total SUDC visits	2.79 (±2.38)	0.00	t= 5.849 (<0.001) *	Not computable
Duration of stay in hospital	22.95 (±8.30)	21.40 (±9.17)	t= 0.803 (0.424)	NA
Number of group therapy sessions	3.64 (±2.28)	2.68 (±1.95)	t= 1.893 (0.061)	NA
Number of individual therapy sessions	2.94 (±1.48)	1.80 (±1.44)	t= 3.402 (0.001) *	d= 0.780

**Table 3:** Comparison of follow-up craving indices between abstinent and relapsed users *p<0.05 statistically significant; **missing data: two in the abstinent group and 17 in the relapsed group; ACQ: alcohol craving questionnaire; CI: craving index; NA: not applicable

Parameter	Abstinent (n=83), Frequency (%) or Mean (SD)	Relapsed (n=8), Frequency (%) or Mean (SD)	Between - subjects effects	Effect size
			F statistic (p-value)	
ACQ				
Baseline	35.10 (±18.44)	36.13 (±20.52)	F=11.742 (p < 0.001)	η² _p _= 0.117
Week 2	23.25 (±15.05)	8.25 (±11.72)		
Week 4**	20.90 (±12.81)	0.00		
Week 8**	20.00 (±12.15)	0.00		
Week 12**	19.30 (±11.18)	0.00		
Craving Index (CI)				
Baseline	2.92 (±1.54)	3.01 (±1.71)	F=0.885 (p = 0.349)	η² _p _= NA
Week 2	1.93 (±1.26)	0.69 (±0.97)		
Week 4**	1.73 (±1.05)	0.00		
Week 8**	1.67 (±1.02)	0.00		
Week 12**	4.48 (±26.29)	0.00		

Further analyses

Among the relapsed users, a positive history of substance abuse in the family was associated with early onset of alcohol use and becoming dependent on alcohol at a younger age in the patients. Also, a positive history of completed suicide in the family was associated with increased amounts of alcohol use per day.

Correlation analyses revealed that younger age of first use of alcohol and earlier use of alcohol was associated with increased impulsivity in attention and motor facet domains. The younger age of initiation of alcohol use was associated with greater levels of psychoticism and antagonism. Alcohol use severity at baseline was predictive of craving towards alcohol during follow-up visits, as indicated by a significant positive correlation between baseline SADQ score and follow-up ACQ and CI scores.

The young age of initiation of alcohol use was associated with increased craving for alcohol at baseline, as indicated by a negative correlation between the age of first use and baseline craving index. A positive correlation was found between the age of first use of alcohol and the CI score at 12 weeks. This is shown in Table 4 and Table 5.

**Table 4:** Correlation between alcohol usage characteristics and severity of alcohol use disorder ACQ: alcohol craving questionnaire; CI: craving index; SAD-Q: severity of alcohol dependence questionnaire; IU: international unit

Parameter	Age of first use (in years) Pearson’s r (p-value)	Age of dependence (in years) Pearson’s r (p-value)	Time to dependence (in years) Pearson’s r (p-value)	IU per day Pearson’s r (p-value)
ACQ Baseline	-0.244 (0.010) *	-0.105 (0.275)	0.139 (0.147)	-0.072 (0.454)
CI Baseline	-0.237 (0.013) *	-0.105 (0.275)	-0.141 (0.142)	-0.068 (0.479)
SAD-Q	-0.097 (0.311)	-0.101 (0.294)	-0.021 (0.824)	-0.004 (0.970)

**Table 5:** Correlational analysis between severity of alcohol use disorder and craving during follow-up *p<0.05 statistically significant; **missing values - 17; ***missing values-18; #missing values - 19; ACQ: alcohol craving questionnaire; CI: craving index; SAD-Q: severity of alcohol dependence questionnaire

Variables	ACQ Baseline Pearson’s r (p-value)	CI Baseline Pearson’s r (p-value)	SAD-Q Pearson’s r (p-value)
ACQ			
2 weeks	0.544 (<0.001) *	0.543 (<0.001) *	0.630 (<0.001) *
4 weeks**	0.416 (<0.001) *	0.415 (<0.001) *	0.624 (<0.001) *
8 weeks***	0.381 (<0.001) *	0.380 (<0.001) *	0.630 (<0.001) *
12 weeks^#^	0.341 (<0.001) *	0.340 (<0.001) *	0.607 (<0.001) *
Craving Index			
2 weeks	0.473 (<0.001) *	0.474 (<0.001) *	0.604 (<0.001) *
4 weeks**	0.415 (<0.001) *	0.413 (<0.001) *	0.624 (<0.001) *
8 weeks***	0.376 (<0.001) *	0.375 (<0.001) *	0.620 (<0.001) *
12 weeks^#^	0.035 (0.740)	0.035 (0.740)	0.170 (0.108)

## Discussion

The present follow-up study aimed to assess the principal factors that influence the treatment outcome, follow-up, and abstinence rates among patients with alcohol use disorder at the time of admission and three months post-discharge. The follow-up duration was taken as three months due to the reasons such as: a) published literature from similar demographic zones which revealed 85% of patients with alcohol use disorder relapsed by 3 months, b) a short follow-up period to ensure the maximum number of participants in the follow-up and to minimize attrition, and c) to minimize logistic difficulties in sustaining long follow-up periods due to COVID-related movement restrictions encountered by the study participants [12,13].

Influence of socio-demographic factors, marriage, and family on abstinence from alcohol

The majority of our patients were males who belonged to the 41-50 years age group. The majority of studies reported in the literature reveal that substance abuse, including alcohol use, is more common among males [14-16]. The age group distribution in our sample was supported by the observations in some of the previous studies, where the majority of patients with alcohol use disorder in hospital settings belonged to the age group of 40-45 years [16,17].

In our study, two-thirds of the sample were married, which is similar to the previous studies reported in the literature [2,15,17]. Studies assert that chronic alcohol use is associated with marital conflicts [18-20]. Compared to the relapsed users, there were more frequent marital conflicts among the abstinent group than the relapsed users. This finding contrasts with the previous findings reported in the literature [18-20]. The discrepancy can probably be due to the majority of married men being older and possibly the frequency of marital conflicts was lesser in older married men. Future studies need to explore the effects of staying abstinent on marital stress and conflicts.

A positive family history of substance abuse was found in around two-thirds of the sample, indicating a high genetic loading of addiction tendencies in our sample. This observation is similar to those found in previous studies [2,16]. The present study also found that a positive family history of substance abuse or suicide was closely linked to early onset, early dependence, and increased amounts of use per day, leading to increased morbidity due to alcohol use. Most previous studies have supported these observations of the influence of a family history of substance use and suicidal behaviors on the onset and evolution of alcohol use disorders among patients [2,16].

Alcohol usage characteristics and abstinence from alcohol

The present study found that most patients tend to initiate around 20 years of age. As noted in previous studies, in our study too, most patients were found to become dependent when they reach their mid-twenties [21,22]. The average time taken to develop physiological dependence was around 5.9 years.

In our study, the majority of the relapsed users relapsed before four weeks of follow-up. At the end of the follow-up period (12 weeks), more than two-thirds of the sample (77.27 %) remained abstinent for the duration of 12 weeks. Such high rates of abstinence among patients with alcohol use disorder can be due to multiple patient-related, illness-related, caregiver-related, and other factors [16].

Among the abstinent group, who maintained abstinence from baseline to the 12 weeks of the follow-up period, the levels of subjective craving towards alcohol at baseline were closely linked to that reported during follow-up. This indicates that baseline severity of craving will be a good measure to predict follow-up abstinence and relapse behaviours of a patient. Previous studies concord with such observations of this study [23].

Psychological attributes and abstinence from alcohol

Early initiation of alcohol use and becoming dependent on alcohol during the early years of life were associated with increased impulsivity among the patients. This has been supported by previous studies, which revealed that negative traits such as impulsivity and hostility were associated with early-onset alcohol use disorder [24-26].

The study found early age of dependence was associated with increased antagonism and psychoticism in the sample. This might possibly explain the reason behind the origin of increased hostility and paranoid ideations among chronic users of alcohol. Similar findings have been noted in previous studies where alcohol use was associated with externalizing personality disorders [27,28]. In contrast to the existing literature, the present study found that the premorbid personality characteristics did not influence the abstinent status during follow-up [29]. This could possibly be due to short duration of follow-up and small sample size.

Treatment compliance

Compared to individual therapy sessions, most patients had attended group therapy sessions more frequently. In addition, the abstinent group were more adherent towards attending both individual and group therapy sessions when compared to the relapsed users. Dose-effect model states there is a positive association between outcome and dose (that is, the number of sessions) but at higher doses the benefit of additional sessions decreases [5,17].

Strengths and limitations

Unlike the previous studies, which have focused on isolated factors, the present study takes an integrated view of all the significant factors that contribute to abstinence. However, the study had a few limitations. The sample size taken for the present study was smaller when compared to the previous studies. The duration of follow-up is shorter in the present study. The observations of the present study conducted in a hospital setting may not be generalizable for community-based rehabilitation interventions. Further moderators of alcohol abstinence, such as caregiver stress, perceived stigma, perceived social support, and intrinsic motivation of the patient, need to be studied in future studies.

## Conclusions

We conclude that patients who remained abstinent throughout the three-month follow-up period displayed certain unique characteristics: they had reported reduced subjective craving towards alcohol at baseline and were more adherent in attending individual and group therapy sessions. Among the relapsed users, most patients relapsed before four weeks. Most of the relapsed patients had a family history of alcohol dependence, psychosis, and suicide. Such factors indicate that high genetic loading of substance abuse led to early initiation and early onset of alcohol dependence.

The effects of genetic loading on alcohol use disorder could be mediated by traits of high impulsivity, psychoticism, and antagonism as suggested by the findings from the present study. A detailed assessment of premorbid personality traits, including impulsivity, psychoticism, and antagonism, may be indicated in persons with early-onset alcohol dependence and in those with frequent relapses of alcohol dependence.

Nevertheless, the present study adds to the literature that baseline assessment of craving is necessary for any patient with alcohol use disorder, which will be a good predictor for craving and abstinence during follow-up.
